# Disordered eating concerns, behaviors, and severity in young adults clustered by anxiety and depression

**DOI:** 10.1002/brb3.2367

**Published:** 2021-11-26

**Authors:** Kaitlyn M. Eck, Carol Byrd‐Bredbenner

**Affiliations:** ^1^ Research Program Coordinator Department of Nutritional Sciences Rutgers University New Brunswick New Jersey USA; ^2^ Department of Nutritional Sciences Distinguished Professor Rutgers University New Brunswick New Jersey USA

**Keywords:** anxiety, cluster analysis, depression, feeding and eating disorders, feeding behavior, major depressive disorder, young adults

## Abstract

**Objectives:**

Little is known about how anxiety and depression in combination relate to eating disorder concerns (eating, shape, and weight concern) and behaviors (restraint eating, binge eating, and purging) indicative of eating disorder symptom severity. This study examined links among disordered eating concerns, behaviors, and severity clustered by depression and anxiety.

**Methods:**

College students (*n* = 1792) completed a survey comprised of the Generalized Anxiety Disorder Scale (GAD‐7), 2‐item Patient Health Questionnaire (PHQ‐2) assessing Major Depressive Disorder (MDD), and Eating Disorder Exam Questionnaire (EDE‐Q) assessing concerns and behaviors indicative of disordered eating.

**Results:**

Cluster analysis yielded four groups: not depressed or anxious to subclinical, moderate, and high depression and anxiety. Analysis of variance (ANOVA) indicated overall eating disorder severity scores increased significantly as GAD and MDD increased, suggesting that as anxiety and depression rise in tandem, disturbed eating severity rises. Results revealed that even at subclinical levels, disordered eating concerns, behaviors, and overall severity scores increase.

**Discussion:**

Future interventions aiming to reduce disordered eating in young adults may be strengthened by incorporating depression and anxiety management strategies. A screening for subclinical anxiety and depression (Mixed Anxiety and Depression Disorder [MADD]) may be helpful in providing early intervention to resolve disordered eating behaviors before they become entrenched.

## Abstract 2

Eating disorder severity scores increased significantly as general anxiety disorder and major depressive disorder increased, suggesting that as anxiety and depression rise in tandem, disturbed eating severity rises. Results revealed that even at subclinical levels, disordered eating concerns, behaviors, and overall severity scores increase.

## 1 INTRODUCTION

Eating disorders often begin during the late adolescent and young adult years with the prevalence among college students estimated to be 4% for males and 11%–17% for females (Lipson & Sonneville, 2017). There are numerous negative mental and physical health effects that are associated with eating disorders, including cardiovascular complications, organ failure, and endocrine dysfunction (Jankauskiene, 2012). As a result of these serious complications, eating disorders have a greater mortality risk than any other mental health disease (Arcelus et al., 2011). Early diagnosis and treatment of eating disorders increase the effectiveness of treatment (National Eating Disorder Association, 2018). It is crucial to understand the etiological risk factors influencing disordered eating behaviors to further prevent morbidity and mortality associated with disordered eating behaviors.

Mental health conditions, such as Generalized Anxiety Disorder (GAD) and Major Depressive Depression (MDD), are associated with elevated eating disorder risk (Godart et al., 2015; Turner et al., 2016). In the United States, the rates of anxiety and depression on college campuses exceed rates in the general adult population (Wang et al., 2018). A study of 621 colleges and universities in the United States found that anxiety was the number one reason college students visit mental health counseling centers on campus (48%), followed by stress (39%) and depression (35%) (LeViness et al., 2018). The same study reported that counseling center directors perceived rates of anxiety and depression to be increasing at their institutions (LeViness et al., 2018).

The fifth edition of the Diagnostic and Statistical Manual of Mental Health Disorders (DSM‐5) defines GAD as excessive worry over events or activities (The American Psychiatric Association, 2013). Symptoms of GAD include irritability, impaired concentration, increased muscle aches and soreness, fatigue, and trouble sleeping (Spitzer et al., 2006). MDD is defined in the DSM‐5 as a feeling of sadness, hopelessness or worthlessness, and loss of interest or pleasure in most activities (The American Psychiatric Association, 2013). In addition to the emotional symptoms, individuals with depression may experience physical symptoms including chronic pain and gastrointestinal issues (Trivedi, 2004). While many studies indicate an association between depression and weight gain (Gibson‐Smith et al., 2016; van Strien et al., 2016), there is evidence that in some cases, depression can cause weight loss in the absence of dieting and decreased energy levels, which can coincide with reduced physical activity (Gibson‐Smith et al., 2016; Weissenburger et al., 1986).

Most of those with depression have significant symptoms of anxiety and vice versa. In fact, GAD and MDD are the most likely to co‐occur of all anxiety and mood disorders (Gorman, 1996; Meng & D'Arcy, 2015). Individuals with both depression and anxiety experience greater physical, social, psychological, and workplace impairment than those with just one of these conditions as well experience reduced likelihood of treatment success (Gaspersz et al., 2018; Katon et al., 2010).

Individuals who exhibit symptoms of both anxiety and depression at a subclinical level may be diagnosed with Mixed Anxiety‐Depressive Disorder (MADD) (Kara et al., 2000). Although not included in the DSM‐5, MADD is included in the 11th revision of the International Statistical Classification of Diseases and Related Health Problems, 5th edition (ICD‐11) which defines MADD as occurring when symptoms of anxiety and depression are both present, but neither predominate and neither are severe enough to warrant a diagnosis of anxiety and/or depression when considered separately. MADD is associated with a reduction in health‐related quality of life and impaired daily living skills, similar to clinically diagnosed GAD and MDD (Möller et al., 2016). This suggests that a combination of subclinical anxiety and subclinical depression can have negative effects on health and well‐being. However, the links between MADD and eating disorder risk are unknown.

While the association between overall eating disorder risk and anxiety and depression is relatively well studied, little is known about how GAD, MDD, and MADD relate to specific concerns (eating concern, shape concern, and weight concern) and behaviors (restraint eating, binge eating, and inappropriate weight‐management behaviors) indicative of eating disorder severity. Given the high rates of anxiety and depression in young adults, coupled with their great risk of disordered eating behaviors (Volpe et al., 2016), the goal of the present study was to examine links among depression and anxiety clusters and severity of disordered eating symptoms in this population.

## 2 METHODS

The Institutional Review Board at the authors’ university approved the procedures for this investigation. All participants gave informed consent prior to data collection.

### 2.1 Sample

Young adult undergraduate college students were recruited through electronic and verbal announcements to complete a cross‐sectional, online survey over a 1‐year period beginning in April 2018. To control for effects of age and culture, the sample eligibility criteria were individuals between the ages of 18 and 25 years who had attended high school in the United States. Recruitment materials specified that the survey aimed to increase knowledge of health‐related behaviors of college students would take approximately 20 min to complete and that participants would be eligible to win 1 of 10 $25 gift cards.

### 2.2 Instruments

The survey gathered demographic data and assessed anxiety and depression as well as disordered eating concerns and behaviors. The Generalized Anxiety Disorder Scale (GAD‐7), which identifies GAD as defined by the DSM‐5, was used to assess anxiety (Spitzer et al., 2006). Scores for this valid, reliable, 7‐item, 4‐point Likert scale (0 = not at all to 3 = nearly every day) were derived by summing individual item scores. Total scores can range from 0 to 21 (Spitzer et al., 2006).

The 2‐item Patient Health Questionnaire (Kronke et al., 2003) (PHQ‐2) was used to assess MDD (clinical depression) as defined by the DSM‐5 (Spitzer et al., 2006). Items on this valid, reliable scale were scored on a 4‐point Likert scale (0 = not at all, 3 = nearly every day), with scores of each item summed to create a total scale score with a possible score range of 0 –6.

The Eating Disorder Exam Questionnaire (EDE‐Q) (PhenX Toolkit & RTI International) assessed concerns and behaviors indicative of eating disorders. The response choices for the Eating Concerns (four items), Shape Concerns (eight items), Weight Concerns (five items), and Restraint Eating (five items) subscales were a 7‐point scale (0 = no days, 1 = 1–5 days, 2 = 6–12 days, 3 = 13–15 days, 4 = 16–22 days, 5 = 23–27 days, 6 = everyday) that assessed frequency of eating disorder symptoms. Item scores for each of these subscales were averaged to create individual subscale scores.

The EDE‐Q Binge Eating Behaviors subscale consisted of one item: “over the past 28 days, how many times have you eaten an unusually large amount of food given the circumstances and had a sense of loss of control at the time.” The EDE‐Q Purging Behaviors subscale had three items assessing the frequency of engaging in inappropriate weight control behaviors (i.e., vomiting, laxative use, and excessive exercise) over the past 28 days. Answers for items on both the Binge Eating Behaviors and Purging Behaviors subscales ranged from 0 to 41 times. The Purging Behaviors subscale scoring reflected the frequency with which each purging behavior is considered clinically significant; a score of 4 or higher indicated clinical significance. Excessively exercising 20 or more times in the past 28 days is considered clinically significant. (Lavender et al., 2010; Luce et al., 2008). Thus, the excessive exercise item was scored as 0 = no excessive exercise, 1 = excessive exercise 1–5 times, 2 = 6–10 times, 3 = 11–15 times, 4 = 16–20 times, 5 = 21–25 times, and 6 = more than 25 times. Both vomiting and laxatives are considered clinically significant when used four or more times in a 28‐day period (Lavender et al., 2010; Luce et al., 2008). Thus, vomiting and laxative were scored as 0 = no use, 1–5 = used 1–5 times, respectively; and 6 = used 6 or more times.

The overall EDE‐Q eating disorder severity score previously developed by Quick and Byrd‐Bredbenner was calculated (Quick & Byrd‐Bredbenner, 2012). This score differs from the Global EDE‐Q score in that it also includes the purging behavior subscales, and thus, is a more comprehensive measure of disordered eating. In addition, it is tailored to the behaviors of the participants in a particular study because it is based on percentiles of participant scores rather than those of other samples who may differ demographically and/or psychographically. This summary score is calculated by determining the 75th and 90th percentile scores for each of the six subscales for the participants in this study. (These percentiles are the cut off points commonly used to categorize psychological measures, with scores above the 75th percentile generally being considered abnormal; Fairburn, 2008; Lavender et al., 2010; Luce et al., 2008). Subscale scores falling below the 75th percentile were scored as 0, scores between the 75th and 90th percentile were scored as 1, and scores above the 75th percentile were scored as 2. The scores for each of the six subscales were summed to create a total EDE‐Q score with a possible range of 0–12.

### 2.3 Data analysis

Descriptive statistics (i.e., means, SDs, and percentages) were computed to describe the study sample. Cronbach alpha scores were calculated to assess internal consistency of scales.

Cluster analysis was conducted using GAD‐7 and PHQ‐2 scores to merge participants into meaningful groups that maximize within‐group homogeneity and between‐group heterogeneity (Yim & KT, 2015). Initially, Ward's hierarchical cluster analysis was conducted to identify the ideal number of clusters. The scree diagram and agglomeration schedule resulting from the hierarchical analysis was reviewed to identify the “elbow” of the scree plot, or the point at which the difference between coefficients increased drastically. This point is used to determine the ideal number of clusters as it represents the point at which increasing the number of clusters would increase heterogeneity within the clusters (Yim & KT, 2015). K‐means cluster analysis was conducted based on the ideal cluster solution identified via the Ward's analysis. Analysis of variance (ANOVA) with Tukey post hoc tests were conducted to identify significant (*p* < .01 for main effects to minimize risks of type 1 errors; *p* < .05 for post hoc tests) differences in weight‐related concerns and behaviors means and overall eating disorder severity score by cluster. Effect size was calculated using the partial eta‐squared statistic. Effect sizes of 0.01 to <0.06, ≥0.06 to <0.14, and ≥0.14 were considered to be small, medium, and large, respectively (Watson & MRC Cognition and Brain Sciences Unit, 2019). All analyses were completed using SPSS software version 26.0 (IBM Corporation, Chicago, IL, USA).

## 3 RESULTS

A total of 2564 students began the survey, after eliminating duplicate entries (*n* = 150), those who did not finish the survey (*n* = 405), and those not meeting inclusion criteria (i.e., 81 were >25 years old; 126 attended high school outside the United States; and 10 were graduate students), the final analytical sample was 1792 participants (65% female). The largest percentage of participants were white (39%), followed by Asian Indian (21%), Asian (e.g., Japanese, Chinese, and Korean 18%), Hispanic (13%), and Black (6%), and the remaining participants were of mixed heritage. The mean age of participants was 20 ± 1.32 SD years.

### 3.1 Description of clusters

The scree plot and agglomeration schedule from Ward's hierarchical cluster analysis revealed that the distance between coefficients increased dramatically at 1788. Thus, the ideal number of clusters for the K‐means analysis was four (sample size of 1792–1788 = 4 clusters).

The generally accepted diagnostic cut off values for the GAD‐7 (i.e., 10 points; Spitzer et al., 2006) and the PHQ‐2 (i.e., three points; Kronke et al., 2003; Löwe et al., 2005; Pedersen et al., 2009; Smith et al., 2010) were used to guide the development of the descriptors for each k‐means cluster. As shown in Table 1, Cluster 1 had the lowest depression and anxiety mean scores, which were well below the cut off values for both scales and, thus, was labeled as the “not depressed or anxious” cluster. Cluster 2 participants had GAD‐7 and PHQ‐2 mean scores significantly higher than Cluster 1, but below the diagnostic cut off values for both anxiety and depression. Thus, Cluster 2 met the ICD‐11 definition of MADD and were labeled as “mixed depression and anxiety.” Cluster 3 participants had mean scores above the diagnostic threshold values and scored significantly higher than both Clusters 1 and 2 and lower than Cluster 4 on both GAD‐7 and PHQ‐2; accordingly, they were described as “moderate depression and anxiety.” Cluster 4 participants had the highest GAD‐7 and PHQ‐2 mean scores, well above the diagnostic cut off values and significantly higher than all other clusters. They were labeled as “high depression and anxiety.” ANOVA and Tukey post hoc analysis of the clustering variables (i.e., GAD‐7 and PHQ‐2) indicated both scales had significant main effects (*p* < .001) with large effect sizes and all pairwise comparisons (*p* < .05) were significant, hence clusters were unique.

**TABLE 1:** Young adult anxiety and depression mean scores by cluster (*n* = 1792)

Measure	Cronbach's alpha	Not depressed or anxious (*n* = 609) mean ± SD (95% CI)	Mixed depression and anxiety (*n* = 567) mean ± SD (95% CI)	Moderate depression and anxiety (*n* = 371) mean ± SD (95% CI)	High depression and anxiety (*n* = 245) mean ± SD (95% CI)	*Fdf =* 3, 1789	*p;* Tukey post hoc test (partial eta‐squared)
Depression^a^	0.79	0.56 ± 0.83 (0.49–0.62)	1.45 ± 1.17 (1.36–1.55)	2.42 ± 1.15 (2.27–2.57)	3.49 ± 1.63 (3.29–3.70)	400.53	<.0001; abcdef^#^ (0.402)
Anxiety^b^	0.93	1.84 ± 1.45 (1.73–1.96)	6.61 ± 1.32 (6.51–6.72)	12.30 ± 1.69 (12.13–12.47)	18.89 ± 1.92 (18.65–19.13)	8519.98	<.0001; abcdef (0.935)

Abbreviation: CI, confidence interval.

^#^ Pairwise comparisons: a = Cluster 1 differs significantly from Cluster 2, b = Cluster 1 differs significantly from Cluster 3, c = Cluster 1 differs significantly from Cluster 4, d = Cluster 2 differs significantly from Cluster 3, e = Cluster 2 differs significantly from Cluster 4, f = Cluster 3 differs significantly from Cluster 4.

^a^ 2‐item Patient Health Questionnaire (PHQ‐2) (Spitzer et al., 2006). Scored on a 4‐point Likert scale (0 = not at all, 1 = several days, 2 = more than half the days, 3 = nearly every day); possible scale score 0–6.

^b^ 7‐item Generalized Anxiety Disorder Scale (GAD‐7) (Kronke et al., 2003). Scored on a 4‐point Likert scale (0 = not at all, 1 = several days, 2 = more than half the days, 3 = nearly every day); possible scale score 0–21.

### 3.2 Disordered eating concerns, behaviors, and severity by cluster

Comparisons of EDE‐Q subscales and overall EDE‐Q score by cluster are shown in Table 2. ANOVA with Tukey post hoc test revealed that restraint eating, shape concern, weight concern, eating concern, and binge eating mean scores differed significantly for all pairwise cluster comparisons with Cluster 1 (not depressed or anxious) having the lowest scores. Effect sizes were large for shape, weight, and eating concerns scales and small for restraint and binge eating. Scores on all these subscales increased across clusters as depression and anxiety severity rose.

**TABLE 2:** Differences in weight‐related concerns and behaviors and overall eating disorder risk by cluster (*n* = 1792)

EDE‐Q (PhenX Toolkit & RTI International, 2019) scales	Not depressed or anxious (*n* = 609) mean ± SD (95% CI)	Mixed depression and anxiety (*n* = 567) mean ± SD (95% CI)	Moderate depression and anxiety (*n* = 371) mean ± SD (95% CI)	High depression and anxiety (*n* = 245) mean ± SD (95% CI)	*Fdf =* 3, 1789	*p;* Tukey post hoc test (partial eta‐squared)
Restraint eating^a^	2.32 ± 1.30 (2.22–2.43)	2.63 ± 1.43 (2.52–2.75)	2.97 ± 1.51 (2.82–3.13)	3.30 ± 1.71 (3.08–3.51)	32.32	<.0001; abcdef^#^ (0.051)
Shape concern^a^	2.11 ± 1.00 (2.03–2.19)	2.67 ± 1.14 (2.58–2.77)	3.23 ± 1.26 (3.10–3.35)	3.73 ± 1.32 (3.56–3.89)	143.74	<.0001; abcdef (0.194)
Weight concern^a^	2.21 ± 1.30 (2.10–2.31)	2.85 ± 1.47 (2.73–2.97)	3.52 ± 1.66 (3.35–3.69)	4.14 ± 1.79 (3.92–4.37)	119.52	<.0001; abcdef (0.167)
Eating concern^a^	1.54 ± 0.79 (1.48–1.60)	2.01 ± 1.09 (1.92–2.10)	2.58 ± 1.46 (2.43–2.73)	3.28 ± 1.78 (3.06–3.51)	141.37	<.0001; abcdef (0.192)
Binge eating^b^	2.75 ± 2.87 (2.52–2.98)	3.58 ± 3.48 (3.30–3.87)	4.76 ± 4.71 (4.27–5.24)	5.08 ± 5.59 (4.37–5.78)	30.82	<.0001; abcdef (0.049)
Purging^c^	0.22 ± 0.03 (0.17–0.28)	0.27 ± 0.03 (0.21–0.32)	0.46 ± 0.03 (0.39–0.52)	0.43 ± 0.04 (0.35–0.51)	13.41	<.0001; bcde (0.022)
Vomiting^d^	0.04 ± 0.45 (−0.03 to 0.10)	0.10 ± 0.69 (0.04–0.16)	0.20 ± 0.93 (0.12–0.28)	0.30 ± 1.18 (0.21–0.40)	8.18	<.0001; bce (0.014)
Laxatives^d^	0.04 ± 0.032 (−0.03 to 0.10)	0.12 ± 0.03 (0.05–0.18)	0.27 ± 0.04 (0.19–0.35)	0.23 ± 0.05 (0.13–0.33)	8.83	<.0001; bcd (0.015)
Excessive exercise^e^	1.60 ± 1.28 (1.50–1.70)	1.59 ± 1.09 (1.50–1.68)	1.89 ± 1.42 (1.74–2.03)	1.76 ± 1.30 (1.59–1.92)	5.51	.001; bd (0.009)
Eating disorder severity^f^	7.08 ± 1.72 (6.94–7.22)	7.74 ± 2.41 (7.54–7.95)	8.98 ± 3.09 (8.65–9.31)	9.95 ± 3.76 (9.46–10.44)	86.46	<.0001; abcdef (0.127)

Abbreviation: CI, confidence interval.

^#^ Pairwise comparisons: a = Cluster 1 differs significantly from Cluster 2, b = Cluster 1 differs significantly from Cluster 3, c = Cluster 1 differs significantly from Cluster 4, d = Cluster 2 differs significantly from Cluster 3, e = Cluster 2 differs significantly from Cluster 4, f = Cluster 3 differs significantly from Cluster 4.

^a^ Scored on a 7‐point scale (0 = no days, 1 = 1–5 days, 2 = 6–12 days, 3 = 13–15 days, 4 = 16–22 days, 5 = 23–27 days, 6 = everyday); possible score range 0–6.

^b^ Possible score ranges from 0 to 41 times. Higher scores indicate greater frequency of behaviors.

^c^ Possible score ranges from 0 to 18. Higher scores indicate greater frequency of behaviors.

^d^ Scored on a 7‐point scale (1 = no engagement in vomiting or laxative use, 2–6 = use of these behaviors 1–5 times, respectively, 7 = use of behaviors 6 or more times); possible score range 0–6.

^e^ Scored on a 7‐point scale (1 = no engagement in excessive exercise, 2 = 1–5 times, 3 = 6–10 times, 4 = 11–15 times, 5 = 16–20 times, 6 = 21–25 times, 7 = more than 25 times); possible score range 0–6.

^f^ Scores for the 6 EDE‐Q subscales were calculated, those falling below the 75th percentile (of study population) were scored as 0, scores between the 75th and 90th percentiles were scored as 1, and scores above the 75th percentile were scored as 2. The scores of the six subscales were summed to create a total EDE‐Q score with a possible range of 0–12. Higher scores indicate greater eating disorder risk.

Purging behaviors also tended to differ significantly among clusters, though effect sizes were low. For all purging behaviors combined, these behaviors were infrequent but tended to become significantly more frequent as depression and anxiety severity rose. The examination of individual purging behaviors indicates that in Clusters 3 and 4, vomiting tended to be significantly more frequent than in Clusters 1 and 2. Participants in the moderate depression and anxiety Cluster 3 had the highest laxative use and excessive exercise scores and tended to engage in these behaviors significantly more often than those in Clusters 1 and 2. Although these differences were significant, the effect size was small and may indicate limited clinical significance.

When considering overall EDE‐Q scores, significant differences were observed between all pairwise clusters comparisons, with scores increasing significantly from Clusters 1 to 4. Effect size was moderate.

## 4 DISCUSSION

This study examined relationships among disordered eating concerns, behaviors, and severity of young adults by MDD and GAD clusters. Findings indicate overall eating disorder severity increases significantly as GAD and MDD mean scores increased across clusters, which suggests that as anxiety and depression rise in tandem, the severity of disordered eating severity rises. This study also demonstrated that depression and anxiety clusters are associated with each of the individual concerns and behaviors considered when assessing overall eating disorder severity. Further, results revealed that even at subclinical levels (i.e., Cluster 2), disordered eating concerns, behaviors, and overall severity increase.

Previous research demonstrated that anxiety and depression each are independently associated with increased eating disorder risk (Godart et al., 2015; Grilo et al., 2009; Kaye et al., 2004; Pallister & Waller, 2008). In addition, studies indicate that anxiety and depression frequently are comorbid (Brown et al., 2001; Meng & D'Arcy, 2015; Moscati et al., 2016). However, the relationships between disordered eating concerns, behaviors, and risk and comorbid anxiety and depression are understudied (Meng & D'Arcy, 2015; Touchette et al., 2011). The findings of the study suggest that when patients receive a diagnosis of an eating disorder (e.g., anorexia nervosa and binge eating syndrome), anxiety and depression also should be assessed and, when diagnosed, treated. Additionally, the finding that each of the individual concerns (weight, shape, or eating) and behaviors (bingeing, purging, and restraint eating) associated with eating disorders increased as depression and anxiety became more severe suggests that both depression and anxiety should be assessed even when just a single disordered eating concern or behavior is present (Meng & D'Arcy, 2015). Attention to anxiety and depression in disordered eating treatment programs is especially important given the greater physical, social, and psychological impairment that occur when depression and anxiety co‐exist (McIntyre et al., 2011; Tiller, 2013) coupled with the higher mortality risk of eating disorders compared to other mental health diseases (Crow et al., 2009; Sullivan, 1995).

Participants in Clusters 3 and 4—fully one‐third of the participants in this study—had mean scores that reached the threshold for the diagnosis of GAD and MDD, a rate comparable to that reported by others for college students (Beiter et al., 2015; Gorman, 1996; LeViness et al., 2018). This high rate is troubling and underscores the importance of campus‐based programs that provide young adults with therapy along with building coping and stress‐management skill, which can help to reduce or control symptoms of depression and anxiety (Kassymova et al., 2018; Yusufov et al., 2019). The prevalence of GAD and MDD also indicates that nutrition education programs targeting this population likely could improve their effectiveness by incorporating coping and stress‐management strategies into healthy eating messaging. Indeed, young adults report high levels of stress (Holinka, 2015; Kattelmann et al., 2014), a known precursor to anxiety and depression (Meng & D'Arcy, 2015).

The pervasiveness of comorbid depression and anxiety also implies that when treating young adults for depression, anxiety should also be evaluated and vice versa. The finding that disordered eating concerns, behaviors, and overall severity progressively increased as depression and anxiety severity rose supports the suggestion of Touchette et al. (2011) with regard to adolescent girls that when depression or anxiety co‐occur, assessment of disordered eating behaviors is indicated (Kaye et al., 2004). Further, prophylactic measures are indicated even in the absence of an eating disorder diagnosis or when disordered eating is at subclinical levels. Interventions such as these are important to consider given that anxiety and depressive disorders tend to predate the development of eating disorders (Hughes, 2012; Kaye et al., 2004; Skinner et al., 2012).

MADD was not included in DSM‐5, primarily because subclinical GAD and MADD symptoms appear to be transient and resolve themselves within a year (Möller et al., 2016). The findings of this study support the value of a MADD diagnosis vis‐à‐vis early recognition of the potential for coincident disordered eating signs and symptoms in that those with subclinical GAD and MDD scores (Cluster 2) had significantly higher scores on concerns, behaviors, and eating disorder severity than the not depressed or anxious cluster. Thus, refraining from a diagnosis until GAD‐7 and/or PHQ‐2 scores reach threshold levels for a clinical diagnosis likely means the seeds of disordered eating concerns and behaviors are already planted (Touchette et al., 2011). Considering that disordered eating behaviors are associated with subclinical anxiety and depression, intervening in these subclinical populations through education and screening for disordered eating behaviors is likely important. This is especially relevant considering that without prompt treatment, disordered behaviors can become entrenched, requiring longer treatment for recovery and increasing the risk of relapse and death (Meng & D'Arcy, 2015; National Eating Disorder Association, 2018). Additionally, because the direction of causality cannot be inferred from the results of the present study, when disordered eating symptoms (including at a subclinical level) are observed, subsequent mental health screenings are warranted. This supports the suggested guidelines for the design of prevention interventions, such as the Health Body Image Program, which aims to prevent eating disorders by raising awareness and by encouraging the use of mental health resources (Jones et al, 2015).

The findings of the present study must be considered in light of its limitations. The study was cross‐sectional and the sample was from a single university and used self‐report questionnaires. The PHQ‐2, GAD‐7, and EDE‐Q are screening tools that identify symptoms of mental health conditions but cannot be used to diagnose conditions. Another limitation may be the potential effects of unmeasured variables (e.g., weight status, socioeconomic status, and sexual orientation) and suggests the need for further research examining clusters variation across sociodemographic groups. However, the sample was large and diverse in terms of gender distribution and ethnic/racial background. In addition, the questionnaires were valid, reliable, widely used assessments of GAD, MDD, and eating disorder severity, and the statistical analysis procedures employed were robust. Future research with a more geographically diverse sample is needed to confirm the findings reported here. In addition, examining links between GAD and MDD, as well as MADD, on other health behaviors as well as in other population groups is warranted. The cross‐sectional design of the current study limits the ability to discern cause and effect; therefore, future longitudinal or experimental research designs can improve our understanding of the relationships noted in this study.

The results indicate that anxiety and depression clusters are associated with concerns, behaviors, and severity indicative of eating disorders. The findings also suggest that even subclinical levels of anxiety and depression elevate eating disorder severity. Screening for subclinical anxiety and depression, and intervening before symptoms advance to clinically diagnosable anxiety and depression may be beneficial in the prevention and/or treatment of eating disorders. Future programs and interventions aiming to reduce eating disorder severity in young adults may be strengthened by incorporating depression and anxiety management strategies. In addition, a diagnosis of MADD may be helpful in providing early intervention to resolve disordered eating behaviors before they become ingrained and difficult to treat.

## CONFLICT OF INTEREST

Kaitlyn M. Eck is currently Employed at Marywood University, 2300 Adams Ave. Scranton, PA 18509. The other author declares no conflict of interest.

## AUTHOR CONTRIBUTIONS

Kaitlyn M. Eck and Carol Byrd‐Bredbenner both participated in the development of the study design, drafting and revising the manuscript, and both authors approved of the final manuscript.

### PEER REVIEW

The peer review history for this article is available at https://publons.com/publon/10.1002/brb3.2367

## Data Availability

The data used in the current study are available from the corresponding author upon request.
